# Biosynthetic Gene Clusters in Sequenced Genomes of Four Contrasting Rhizobacteria in Phytopathogen Inhibition and Interaction with Capsicum annuum Roots

**DOI:** 10.1128/spectrum.03072-22

**Published:** 2023-05-24

**Authors:** Yumiko De la Cruz-Rodríguez, Jesús Adrián-López, Jazmín Martínez-López, Bibiana Itzel Neri-Márquez, Ernesto García-Pineda, Alejandro Alvarado-Gutiérrez, Saúl Fraire-Velázquez

**Affiliations:** a Lab. Biología Integrativa de Plantas y Microorganismos, Unidad Académica de Ciencias Biológicas, Universidad Autónoma de Zacatecas, Zacatecas, Mexico; b Lab. MicroRNAs y Cáncer, Unidad Académica de Ciencias Biológicas, Universidad Autónoma de Zacatecas, Zacatecas, Mexico; c Universidad Michoacana de San Nicolás de Hidalgo, Michoacán, Mexico; Pennsylvania State University

**Keywords:** biosynthetic gene clusters, *Bacillus velezensis*, biocontrol of phytopathogens, antibiotics, secondary metabolites

## Abstract

Through screening of rhizobacteria, species that effectively suppress phytopathogens and/or promote plant growth are found. Genome sequencing is a crucial step in obtaining a complete characterization of microorganisms for biotechnological applications. This study aimed to sequence the genomes of four rhizobacteria that differ in their inhibition of four root pathogens and in their interaction with chili pepper roots to identify the species and analyze differences in the biosynthetic gene clusters (BGCs) for antibiotic metabolites and to determine possible phenotype-genotype correlations. Results from sequencing and genome alignment identified two bacteria as Paenibacillus polymyxa, one as Kocuria polaris, and one that was previously sequenced as Bacillus velezensis. Analysis with antiSMASH and PRISM tools showed that *B. velezensis* 2A-2B, the strain with the best performance of referred characteristics, had 13 BGCs, including those related to surfactin, fengycin, and macrolactin, not shared with the other bacteria, whereas *P. polymyxa* 2A-2A and 3A-25AI, with up to 31 BGCs, showed lower pathogen inhibition and plant hostility; *K. polaris* showed the least antifungal capacity. *P. polymyxa* and *B. velezensis* had the highest number of BGCs for nonribosomal peptides and polyketides. In conclusion, the 13 BGCs in the genome of *B. velezensis* 2A-2B that were not present in the other bacteria could explain its effective antifungal capacity and could also contribute to its friendly interaction with chili pepper roots. The high number of other BGCs for nonribosomal peptides and polyketide shared by the four bacteria contributed much less to phenotypic differences.

**IMPORTANCE** To advance the characterization of a microorganism as a biocontrol agent against phytopathogens, it is highly recommended to analyze the potential of the profile of secondary metabolites as antibiotics that it produces to counteract pathogens. Some specific metabolites have positive impacts in plants. By analyzing sequenced genomes with bioinformatic tools, such as antiSMASH and PRISM, outstanding bacterial strains with high potential to inhibit phytopathogens and/or promote plant growth can be quickly selected to confirm and expand our knowledge of BGCs of great value in phytopathology.

## INTRODUCTION

In the rhizosphere, a variety of microorganisms, including bacteria, collaborate with plants to counteract phytopathogens, take up nutrients through the root system, and promote plant growth. Studies of complex plant-microorganism interactions, including the rhizosphere niche, have only recently begun; the molecular mechanisms that govern these interactions are still unclear. In modern agriculture, the use of microorganisms with phytopathogen-inhibiting and/or growth-promoting properties in plants is increasing, and the positive impact of microorganism-plant interaction on the nutritional value of the harvested product is also considered, giving rise to the new concept “nutrition-sensitive agriculture” (1, 2).

The most complete characterization of microorganisms for biotechnological application demands knowledge of the information in their genomes. Genomic sequencing together with bioinformatic tools allows us to perform genome mining to decipher the molecular mechanisms that microorganisms develop to perform specific biological functions. Secondary metabolites produced by microorganisms are not directly involved in their growth or reproduction but in some cases provide benefits to the producer by acting as inhibitors against other competing microorganisms, such as bacteria, fungi (3, 4), and insects (5). Biosynthetic gene clusters (BGCs) are groups of genes in a genome involved in an enzymatic pathway for the biosynthesis of natural products with diverse chemical structures and biological functions and often provide advantages to the microorganism by improving their fitness in the habitat. Natural products, such as secondary metabolites, are used as weapons to fight and eradicate other contending organisms (6). Structural classification of BGCs includes nonribosomal peptide synthetases (NRPS), polyketide synthases (PKS), lanthipeptides, and bacteriocins. NRPS and PKS are related to the synthesis of antibiotics and other pharmaceutical products (7, 8). With genomic sequencing and bioinformatic tools, the analysis and determination of BGCs in newly explored genomes allow for the identification of bioactive compounds that a microorganism can produce, even when the production process cannot be established *in vitro* or the microorganism is difficult to cultivate. Horizontal gene transfer is a major source of evolution and diversity of BGCs, and the exchange of BGCs occurs easily in bacteria (9). Genome mining analysis in bacteria has revealed that BGCs exhibit high diversity and variable distribution within bacterial species, even in closely related strains, to such an extent that strains of the same species can present high variation in the BGCs that they contain in their genomes (8). The objective of this study was to sequence the genome of four rhizobacteria with contrasting phenotype in the inhibition of phytopathogens and interaction with roots of chili peppers (Capsicum annuum). Genome sequencing, assembly, alignment, species determination, identification of BGCs, and determination of similarities and differences in the content of BGCs in bacteria were performed, and the correlation of BGCs with the known phenotypes of these microorganisms was established.

## RESULTS

### Inhibition of pathogens by bacterial strains and behavior of bacterial strains in plant roots.

**FIG 1:**
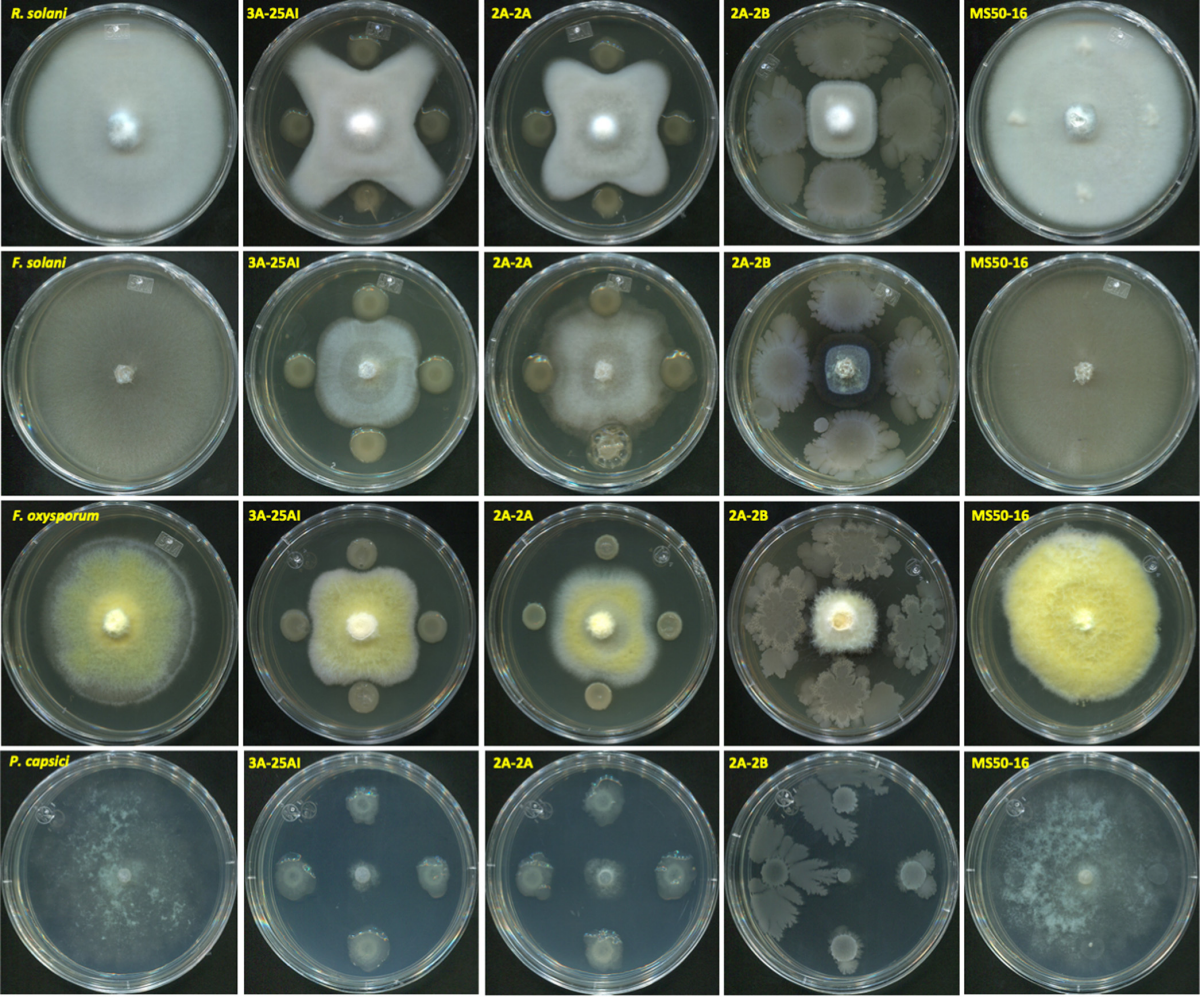
Bacteria and phytopathogens dual confrontations in PDA medium after 6 to 12 days of bacterial growth and 7 to 14 days of pathogen growth. In the top row, the pathogen Rhizoctonia solani without contact (1st column) or in contact with, from left to right (2nd to 5th column), 3A-25AI, 2A-2A, 2A-2B, and MS50-16 bacterial strains. In the second row, the pathogen Fusarium solani in contact with the same bacteria and the same order described above. In the third row, the pathogen Fusarium oxysporum in contact with the same bacteria and the same order. In the bottom row, the pathogen *Phytophthora capsici* in contact with the same bacteria.

**FIG 2:**
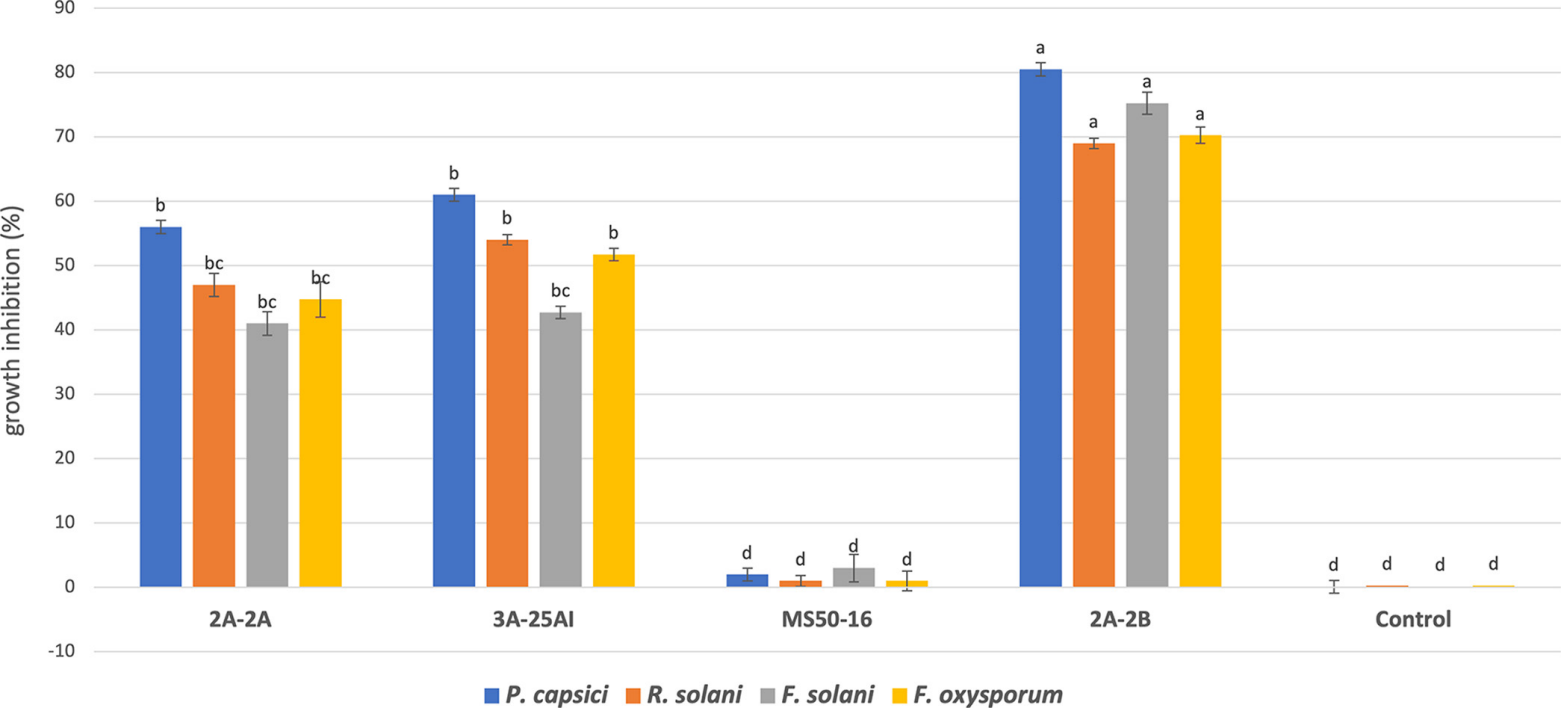
Growth inhibition of four root pathogens in pepper plant by four bacterial strains from the rhizosphere or soil. Pathogens include *Phytophthora capsici*, Rhizoctonia solani, Fusarium solani, and Fusarium oxysporum. ANOVA statistical analysis and Tukey test were performed.

The strain 2A-2B, in a dual confrontation in Petri dishes in three culture media (peptone-dextrose agar [PDA], Trypticase soy agar [TSA], and King B [KB]), showed between 68 and 81% growth inhibition of virulent strains of the fungal pathogens Fusarium solani, Fusarium oxysporum, and Rhizoctonia solani and the oomycete pathogen Phytophthora capsici in chili peppers (Fig. 1 and 2). Bacterial strains 2A-2A and 3A-25AI, inhibited the *in vitro* growth of pathogens at an intermediate level (43 to 61%); while MS50-16 inhibited only between 1 and 3% of pathogen growth (Fig. 1 and 2).

**FIG 3:**
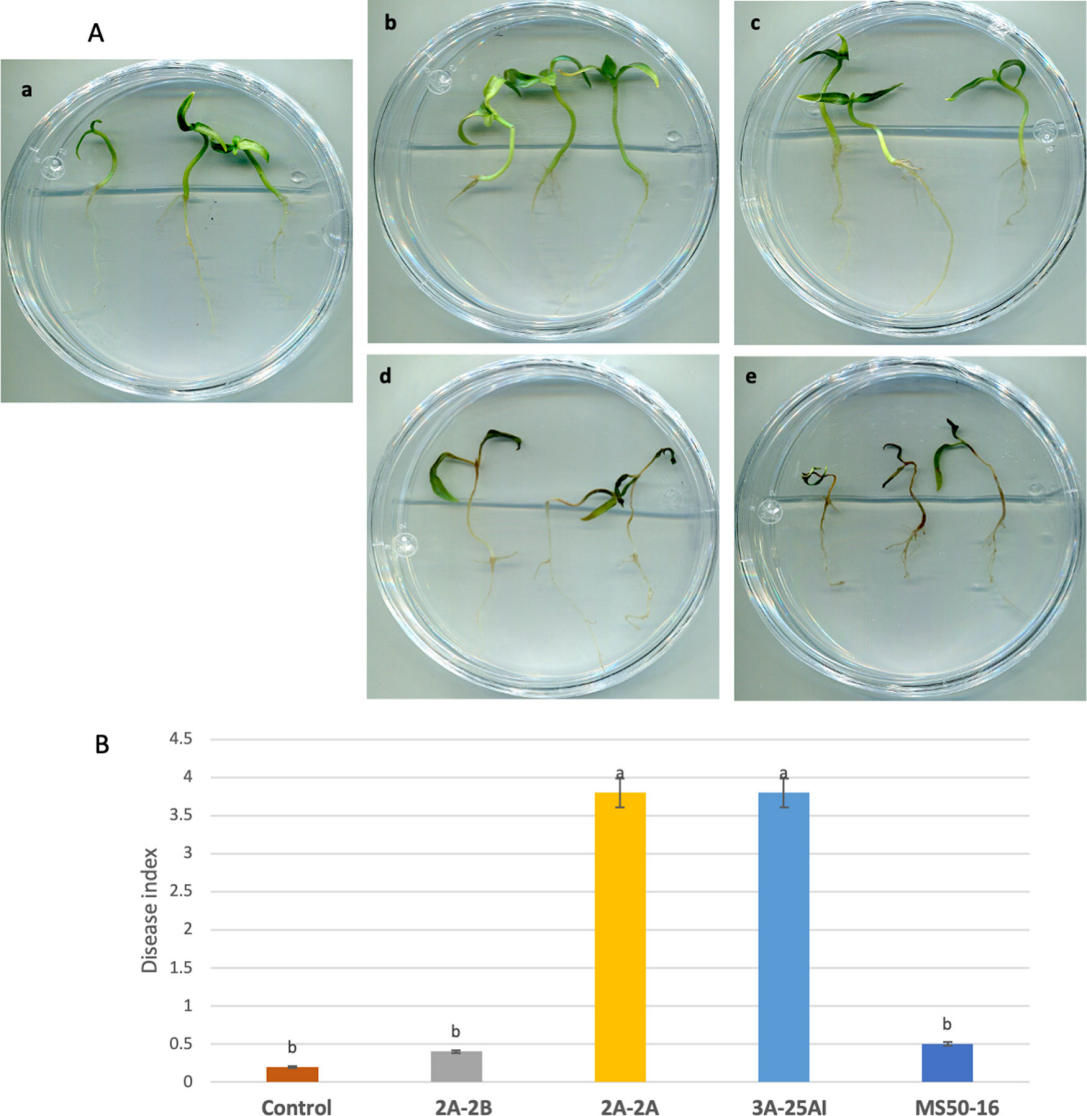
Behavior of bacterial strains when inoculated in roots of 15-day-old chili pepper seedlings, under *in vitro* conditions, and statistical results. Results 6 days postinoculation. (A) Plant-bacteria interactions, control treatment in image a. Friendly plant-bacteria interactions in images b and c; in b, strain 2A-2B; in c, strain MS50-16. Hostile plant-bacteria interactions in images d and e; in d, strain 2A-2A; in e, strain 3A-25AI. (B) ANOVA statistical analysis and Tukey test were performed.

In relation to plant-bacteria interaction, in *in vitro* tests 6 days postinoculation, 2A-2B and MS50-16 behaved harmlessly (friendly) when inoculated in the root of chili pepper seedlings cultured in Petri dish in Murashige and Skoog (MS) medium (Fig. 3), with similar behavior in root inoculation in potted plants (data not shown). Conversely, 2A-2A and 3A-25AI showed hostile behavior and caused necrosis when inoculated in the root of chili pepper seedlings, with a disease severity index (DSI) of 3.7 on a scale of 4, with statistical differences (*P *< 0.05) compared to the control and 2A-2B treatments (Fig. 3A and B).

### Protection in plant against pathogens *P. capsici* and R. solani via root preinoculation with bacteria 2A-2B.

**FIG 4:**
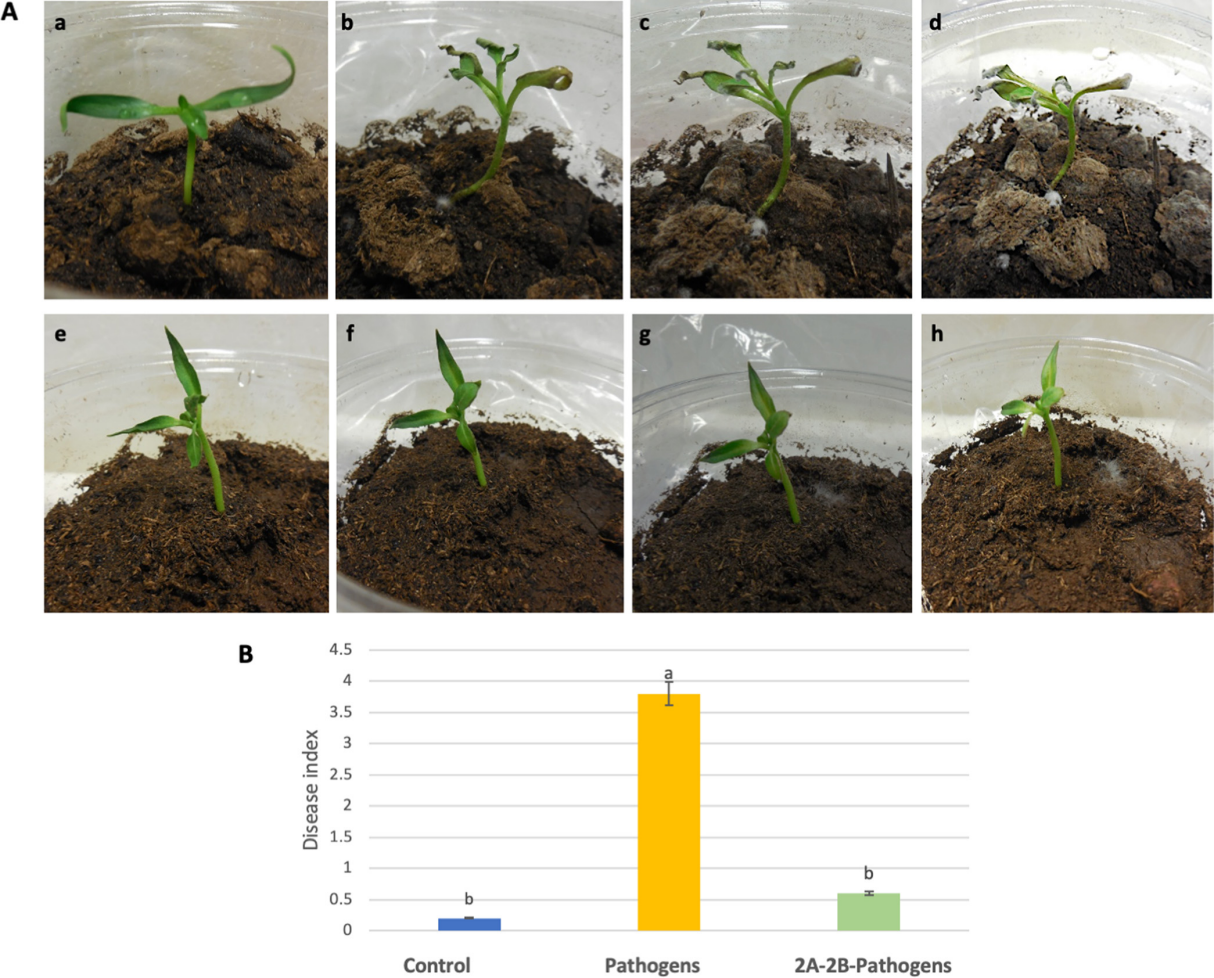
Protection in plant infected with *P. capsici* and R. solani via root preinoculation with bacterial strain 2A-2B. The experiment was conducted in 18-day-old plants and went on for 6 days. (A) The top row shows plants inoculated with pathogens *P. capsici* and R. solani; a, time zero; b, c, and d, 48, 72, and 96 h postinoculation, respectively. (A) The bottom row shows plants preinoculated with bacterial strain 2A-2B and immediately inoculated with strains of the pathogens *P. capsici* and R. solani. The observation times for the images e, f, g, and h were as mentioned above. (B) ANOVA statistical analysis and Tukey test were performed.

Under pot conditions and in bioclimatic chamber, preinoculation in the roots of chili pepper plants with 2A-2B bacteria offered a protective effect against the attack of the pathogens *P. capsici* and R. solani. Plants preinoculated with bacteria and then with pathogens had a disease index of 0.6 at 4 days postinoculation, while the disease index increased in plants without preinoculation with bacteria and inoculated with pathogens to 3.8 on a scale of 4, with statistical differences (*P* < 0.05) (Fig. 4).

### Genomic sequences and their distinctive features in bacterial strains 3A-25AI, 2A-2A, 2A-2B, and MS-5016.

In the assemblies with SPAdes software (10), partially sequenced genomes (draft) with variable sizes were achieved. The genome of MS50-16 was the smallest strain with a length of 3.43 MB and had 3,164 genes and an *N*_50_ of 136. The genome of 2A-2B was the intermediate size of 3.96 MB, containing 3,962 genes and an *N*_50_ of 64. The largest genomes were 2A-2A and 3A-25AI, with 5.64 and 5.60 MB, respectively; 2A-2A and 3A-25AI had 5,056 and 5,490 genes and *N*_50_ values were 190 and 100, respectively. The percentage of GC content was 46% for the genome of 2A-2B, 65.5% for MS-5016, and 45.5% for 2A-2A and 3A-25AI (Table 1). From the annotation of genomes with the Prokka tool (11), the following was defined: 2A-2B contains 3,713 protein-coding genes and 89 RNA genes, 3A-25AI has 4,968 protein-coding genes and 38 RNA genes, 2A-2A contains 5,163 protein-coding genes and 39 RNA genes, and MS50-16 contains 3,116 protein-coding genes and 58 RNA genes.

**TABLE 1:** Basic data and quality parameters for the four sequenced genomes

Bacterial strain	NCBI reference sequence	Length (MB)	Coverage	Total genes	CDSs	*N*_50_	*L*_50_	GC (%)
Bacillus velezensis 2A-2B	NZ_MLCV00000000.1	3.958	38.0×	3,962	3,874	64	5	46.0
Paenibacillus polymyxa 2A-2A	NZ_JALGWU000000000.1	5.641	82.0×	5,056	5,019	190	10	45.5
Paenibacillus polymyxa 3A-25AI	NZ_MLCZ00000000.1	5,602	13.0×	5,490	5,445	100	74	45.5
Kocuria polaris MS50-16	MLDA00000000.1	3,433	25.0×	3,164	3,108	136	39	65.5

### Bacterial species were identified using genome alignment with the prokaryotic data bank in the NCBI GenBank and by average nucleotide identity.

**FIG 5:**
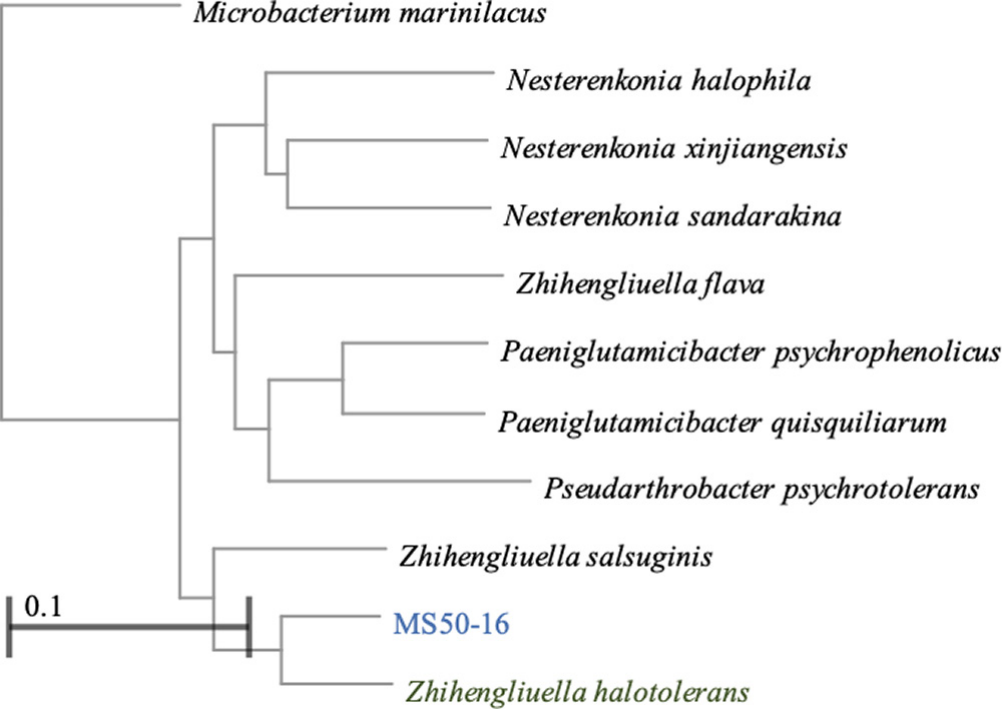
Phylogenetic tree based on the genome of bacterial strain MS50-16 and 10 other accessions with major identity in the NCBI data bank. Phylogenetic tree constructed using Neighbor-joining method.

The genome alignments in the NCBI GenBank were used to elucidate the coverage and identity of the species of bacteria the studied strains belong to. In the case of 2A-2B, the alignment showed high coverage and identity of 96 and 98.4%, respectively, with Bacillus velezensis JS25R (NZ_CP009679.1), which was the closest phylogenetically, with a genome size of 4.00 MB. The genomes of 2A-2A and 3A-25AI aligned with high coverage and identity (100 and 98 to 99%, respectively) with Paenibacillus polymyxa ZF129 (NZ_CP040829.1), which is 5.70 MB. The genome of MS-5016 aligned to Kocuria polaris. When using average nucleotide identity (ANI) to compare two genomes in prokaryotes, an average cutoff greater than 95% indicates that two specimens belong to the same species (12). The OrthoANIu metric (13) was used to compare the genomes downloaded from the NCBI GenBank. The ANI for 2A-2B and Bacillus velezensis JS25R was 98.35%. For 2A-2A and Paenibacillus polymyxa CF05, the ANI was 98.61%. The ANI for 3A-25AI and Paenibacillus polymyxa CF05 was 98.59%. The ANI was less than 85% for MS50-16 and Kocuria polaris. Although the registration of the accession of MS50-16 (MLDA01000000) in the NCBI GenBank was carried out months before, a current review of genome identity yielded the highest identity with Zhinhengliuella halotolerans (NZ_SHLA01000001.1) with 81 and 94.12% coverage and identity, respectively (Fig. 5), while Kocuria turfanensis was further away with 84.9% identity.

The alignments between the genomes of *P. polymyxa* 2A-2A and 3A-25AI suggest that there is a very high level of identity between these strains, with extensive regions of alignment in mostly sense strand DNA and less in the antisense strand. The average identity between these two genomes was 99.96%, although there was also a high number of breakpoints, translocations, insertions, and few relocations (data not shown).

### Large-scale evolutionary events in the genome of *B. velezensis* 2A-2B.

**FIG 6:**
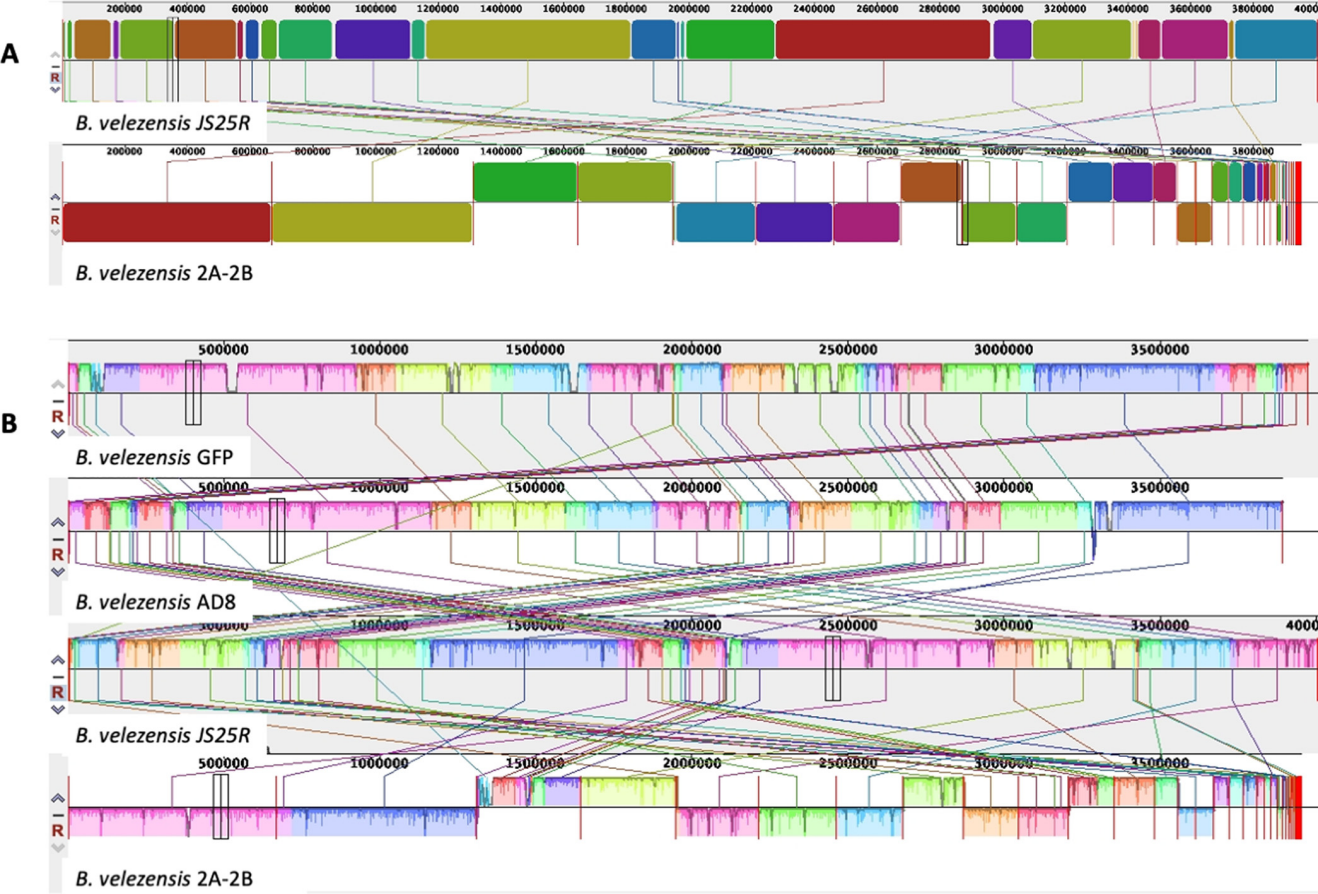
Representation of 28 or more local colinear blocks (LCBs) between chromosomal sequences of Bacillus velezensis 2A-2B and the reference genome *B. velezensis* JS25R, in addition the GFP and AD8 reference genomes from *B. velezensis*. (A) Alignment of bacterial strain 2A-2B and *B. velezensis* JS25R as reference genome. (B) Multiple alignment of genomes between 2A-2B strain with respect to the other three genomes of *B. velezensis* strains with the highest identity in the gene bank. The alignment was performed in Mauve tool, version snapshot 2015 (47).

Local colinear blocks (LCBs) were identified by aligning the genomes of 2A-2B and JS25R from the NCBI GenBank using the Mauve tool (14) in progressive Mauve option to analyze rearrangements in the genome of 2A-2B. There were 24 LCBs with a minimum weight of 9,093. The conserved regions between both genomes cover the entire extension of the *B. velezensis* 2A-2B, with no region lacking homology. In the genome of 2A-2B, at least 12 LCBs were found in the same orientation, and 11 LCBs were found in reverse complement (inverse) orientation with respect to the reference genome (Fig. 6A). The inverted regions in the genome of 2A-2B are a distinctive characteristic of this strain, given that in a multiple alignment in which three other genomes of *B. velezensis* with the highest identity were included, JS25R, AD8, and GFP-2, these rearrangements were observed only in 2A-2B (Fig. 6B).

### Biosynthetic gene clusters for antimicrobial secondary metabolites identified using antiSMASH.

**FIG 7:**
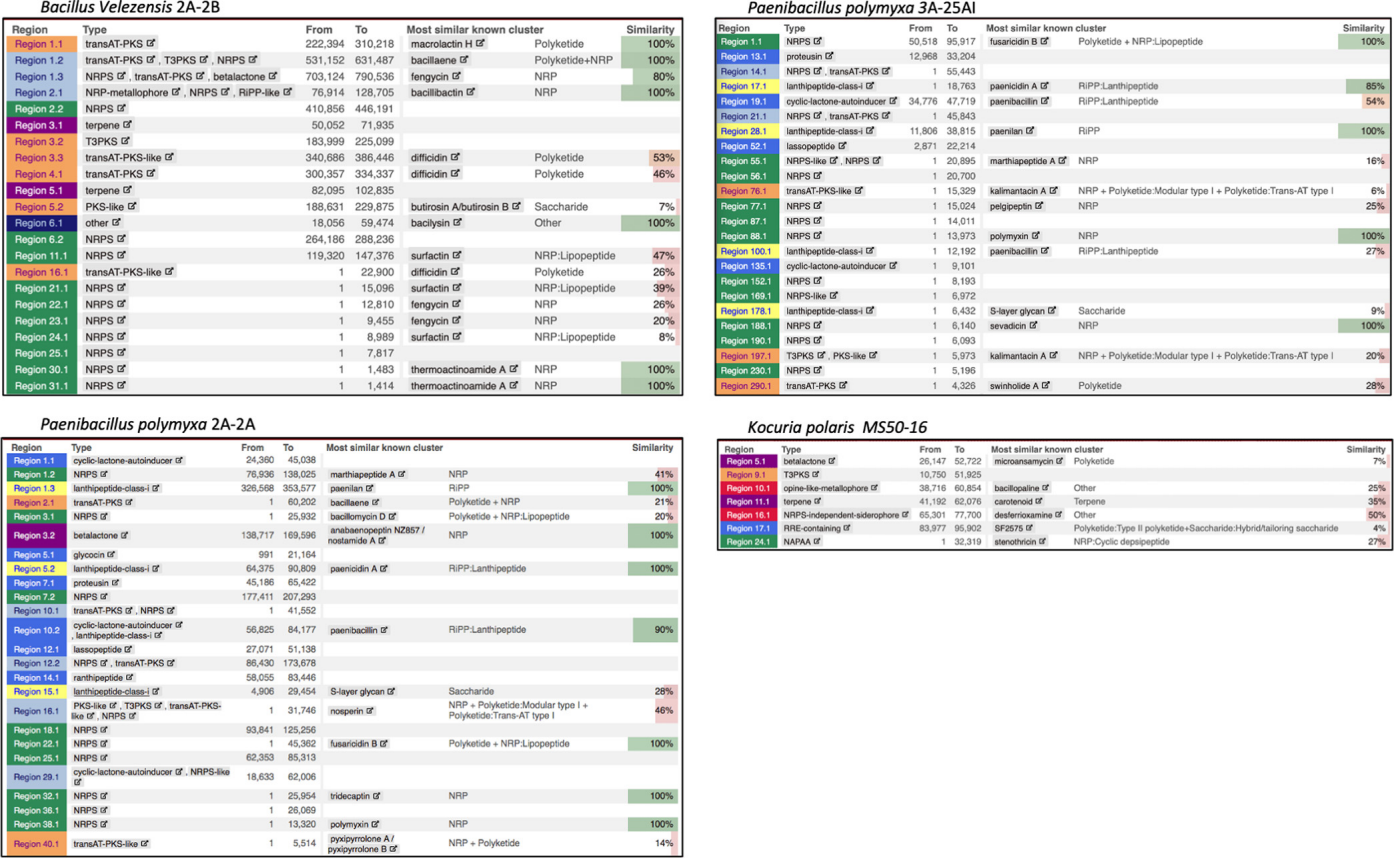
Conserved biosynthetic gene clusters (BGCs) for biosynthesis of secondary metabolites in the genomes of the four bacterial strains studied. In each case, to the right side, in the green background, BGCs with similarity between 80 and 100% and, in red background, BGCs with similarity less than 80%. These data correspond to results of the antiSMASH algorithm analysis.

For *B. velezensis* 2A-2B, the genome analysis in antiSMASH revealed 22 regions containing BGCs for the biosynthesis of antimicrobial peptides, 7 of these with high similarity (80 to 100%) to known clusters, those related to the biosynthesis of macrolactin H, bacillaene, fengycin, bacillibactin, bacilysin, and thermoactinoamide A (2); with less similarity (8 to 53%), those related to biosynthesis of difficidin (3), surfactin (3), fengycin (2), and butirosin A/butirosin B; and six NRPS and PKS types without any similarity to known clusters in the GenBank (Fig. 7). These 22 regions cover a total of 739 kb, which represents 18.6% of the genome.

For *P. polymyxa* 2A-2A, the antiSMASH analysis showed that there were 25 regions of the genome that contain BGCs as follows: with high similarity (90 to 100%) to known clusters, those related to paenilan, anabaenopeptin NZ857/nostamide A, paenicidin A, fusaricidin B, tridecaptin, and plymyxin; with less similarity (14 to 46%) to known genes, those related to marthiapeptide A, bacillaene, bacilomycin D, S-layer glycan, nosperin, and pyxipyrrolone A/pyxipyrrolone B; and 12 BGCs had no similarity to known clusters in the GenBank (Fig. 7). The 25 regions with BGCs were 815 kb, which is 14.4% of the genome.

For the *P. polymyxa* 3A-25AI genome, the antiSMASH analysis showed a somewhat different profile to that of 2A-2A. There were 24 regions with BGCs as follows: 5 showed high similarity to known BGCs, and within these were fusaricidin B, paenicidin A, paenilan, polymyxin, and sevadicin; with less similarity (16 to 54%), those BGCs oriented to the biosynthesis of paenibacillin, marthiapeptide A, kalimantacin A (2), pelgipeptin, S-layer glycan, and swinholide Ax (Fig. 7). The 24 regions with BGCs comprise 4.35 kb, which represents 5.7% of the 3A-25AI genome.

Gene clusters conserved in genomes of both *P. polymyxa* strains for biosynthesis of antibiotic metabolites include seven BGCs for biosynthesis of furacidin B (conserved with 100% similarity in both strains), paenicidin A (conserved with 100 and 85% similarity in 2A-2A and 3A-25AI, respectively), paenilan (100% similarity to known BGCs in both strains), polymyxin (100% similarity to known BGCs in both strains), paenibacillin (one BGC in 2A-2A and two BGCs in 3A-25AI; in 3A-25AI with less similarity to known BGCs), marthiapeptide A, and S-layer glycan. In contrast, divergent BGCs between the genomes of both *P. polymyxa* strains are the BGCs involved in the biosynthesis of bacillaene, bacillomicin D, anabaenopeptin NZ857/nostamine A, nosperin, tridecaptin, and pyxipyrolone A/pyxipyrrolone B, which were found on the side of 2A-2A, and sevadicin, kalimantacin A, pelgipeptin, swinholide, and others still unknown, which were found on the side of 3A-25AI (Fig. 7).

**FIG 8:**
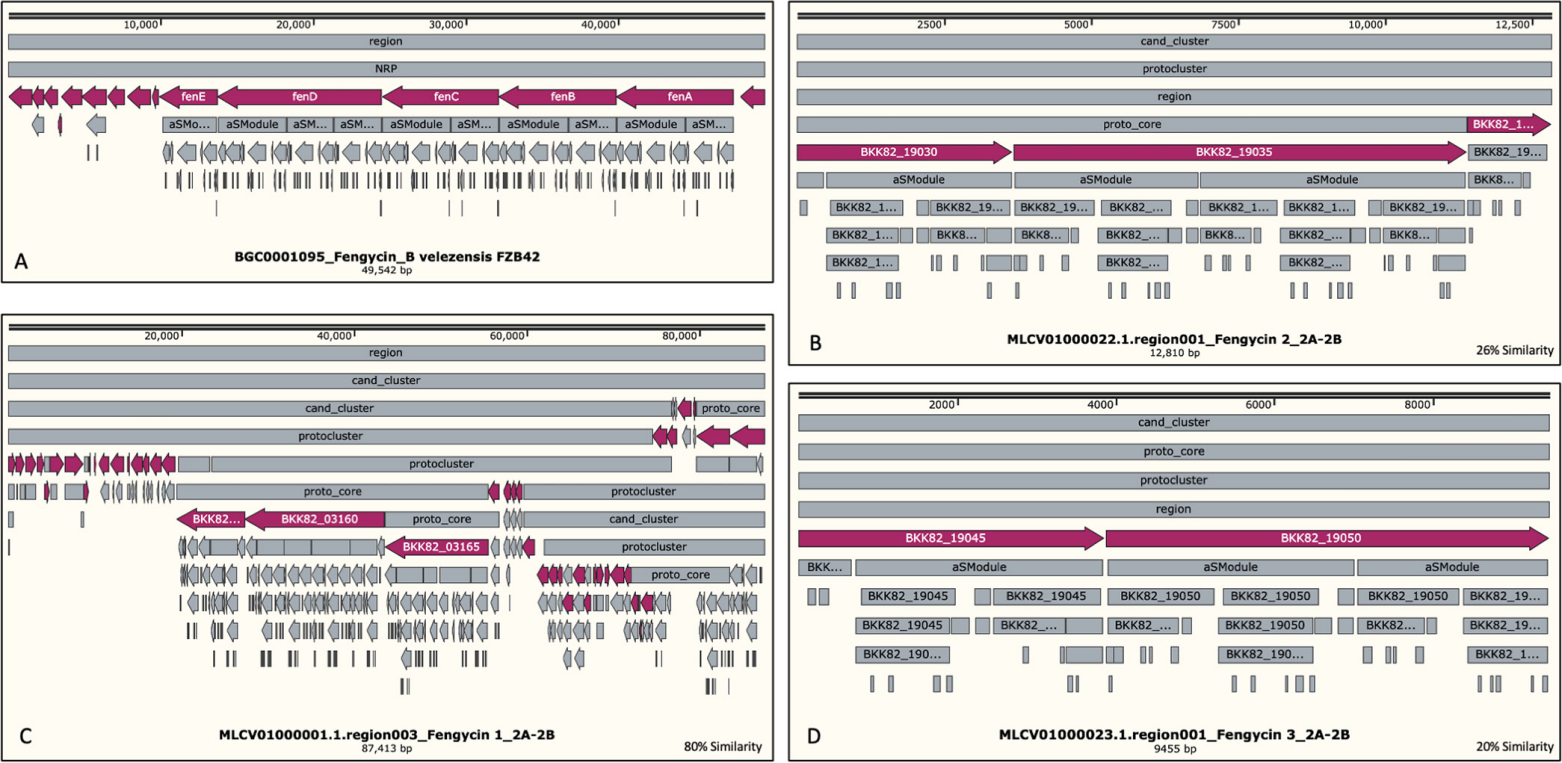
Comparisons of structural arrangement and number of genes in the BGC for fengycin biosynthesis, found in the genomes of bacterial strain 2A-2B and Bacillus velezensis FZB42 as reference BGC, obtained from the repository of BGCs in the MIBiG online server. (A) Well-described 87.4-MB reference BGC for fengycin biosynthesis in *B. velezensis* FZB24; in the red background are five core genes, *fenA* to *fenE*; 10 modules and other regulatory genes are in the gray background. (B, C, and D) BGCs for fengycin biosynthesis in the genome of 2A-2B strain. (B) A 12.8-kb BGC for fengycin biosynthesis with 26% similarity. (C) A 87.4-kb BGC for fengycin with 80% similarity. (D) A 9.4-kb BGC with 20% similarity. This information was obtained using the online antiSMASH tool.

**FIG 9:**
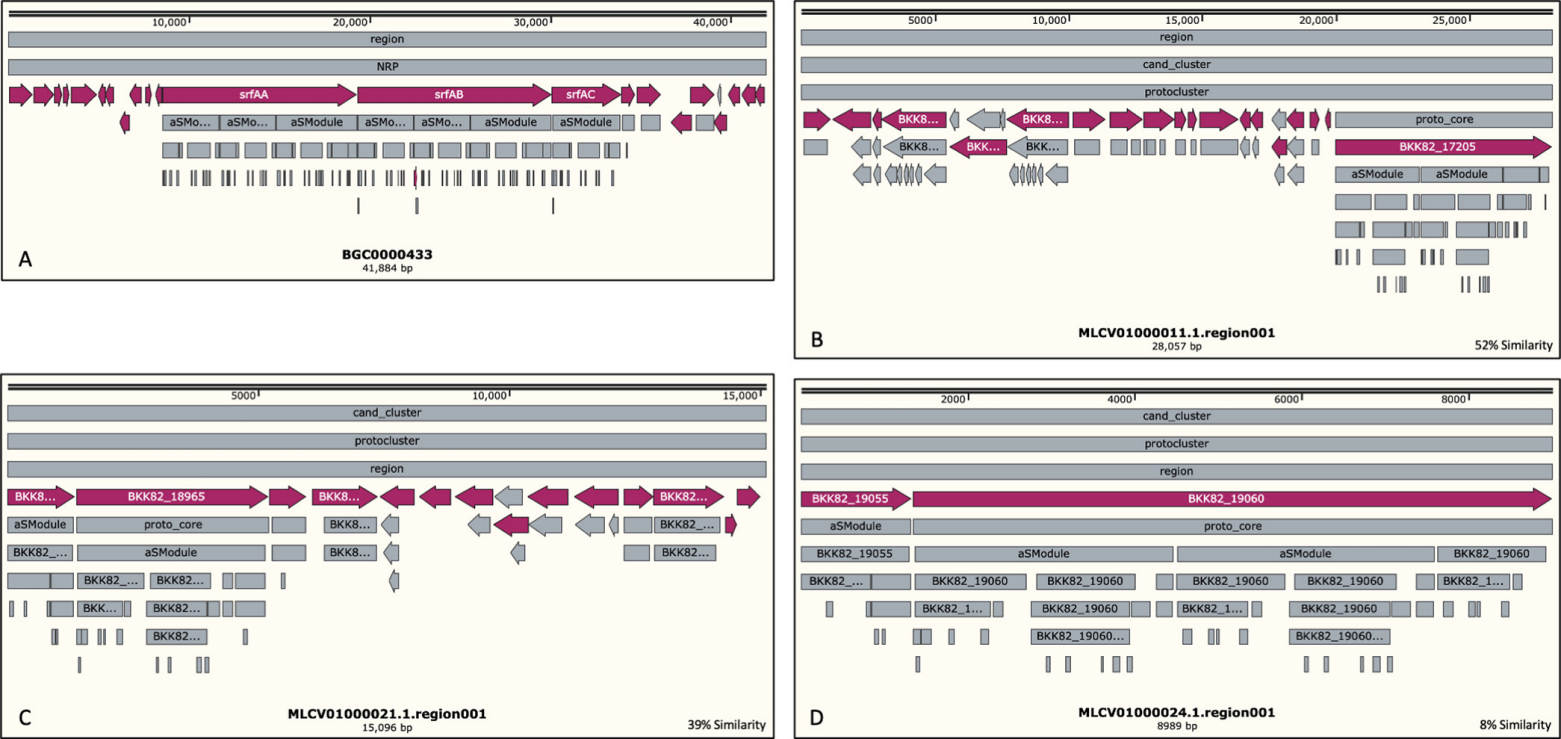
Comparisons of structural arrangement and number of genes in the BGC for surfactin biosynthesis found in genomes of bacterial strain 2A-2B and Bacillus velezensis FZB42 as reference BGC, obtained from the repository of BGCs in the MIBiG online server. (A) Well-described reference 41.8-kb BGC for surfactin in *B. velezensis* FZB24; in red background are three central core genes, *srfAA* to *srfAC* and 19 core genes, and about 30 modules and regulatory genes are in the gray background. (B, C, and D) BGCs for surfactin biosynthesis in the genome of the 2A-2B strain. (B) A BGC for surfactin biosynthesis with 52% similarity. (C) A 2nd BGC for fengycin with 80% similarity. (D) A 3rd BGC with 20% similarity. This information was obtained using the online antiSMASH tool.

**FIG 10:**
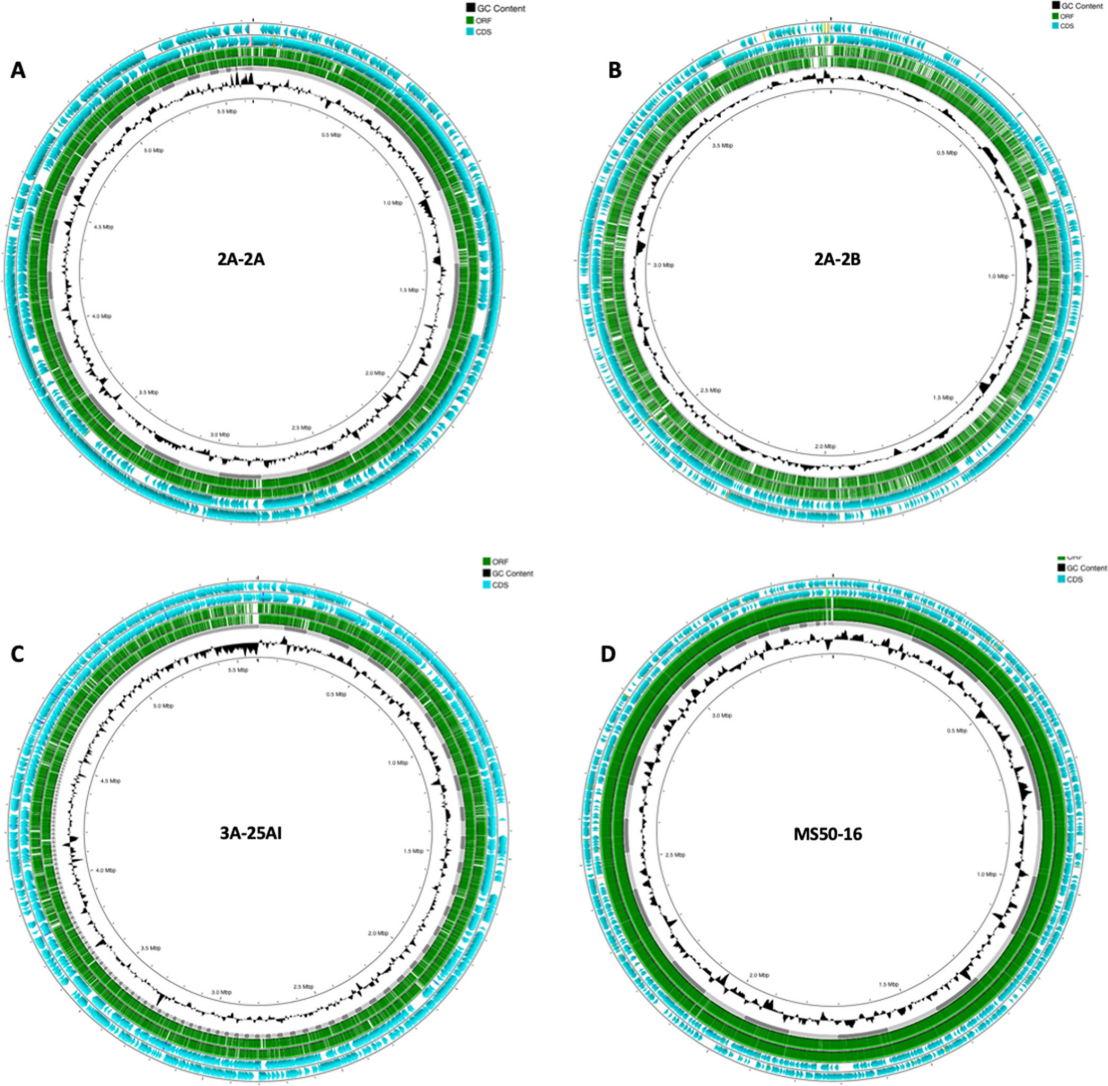
Circular map of genomes of the four bacteria with indications of genome size, GC content, contigs, coding sequences, and open reading frames (ORFs) in the DNA. Inner ring, thousands of bp of the genome; 2nd ring, GC content; 3rd ring, location and size of contigs; 4th and 5th ring, open reading frames (OPF) in sense and antisense DNA strands; 6th and 7th rings, coding sequences on sense and antisense DNA strand. (A) Paenibacillus polymyxa 2A-2A; (B) Bacillus velezensis 2A-2B; (C) *P. polymyxa* 3A-25AI; (D) strain MS-5016. The maps were constructed using the CGView tool (45).

**FIG 11:**
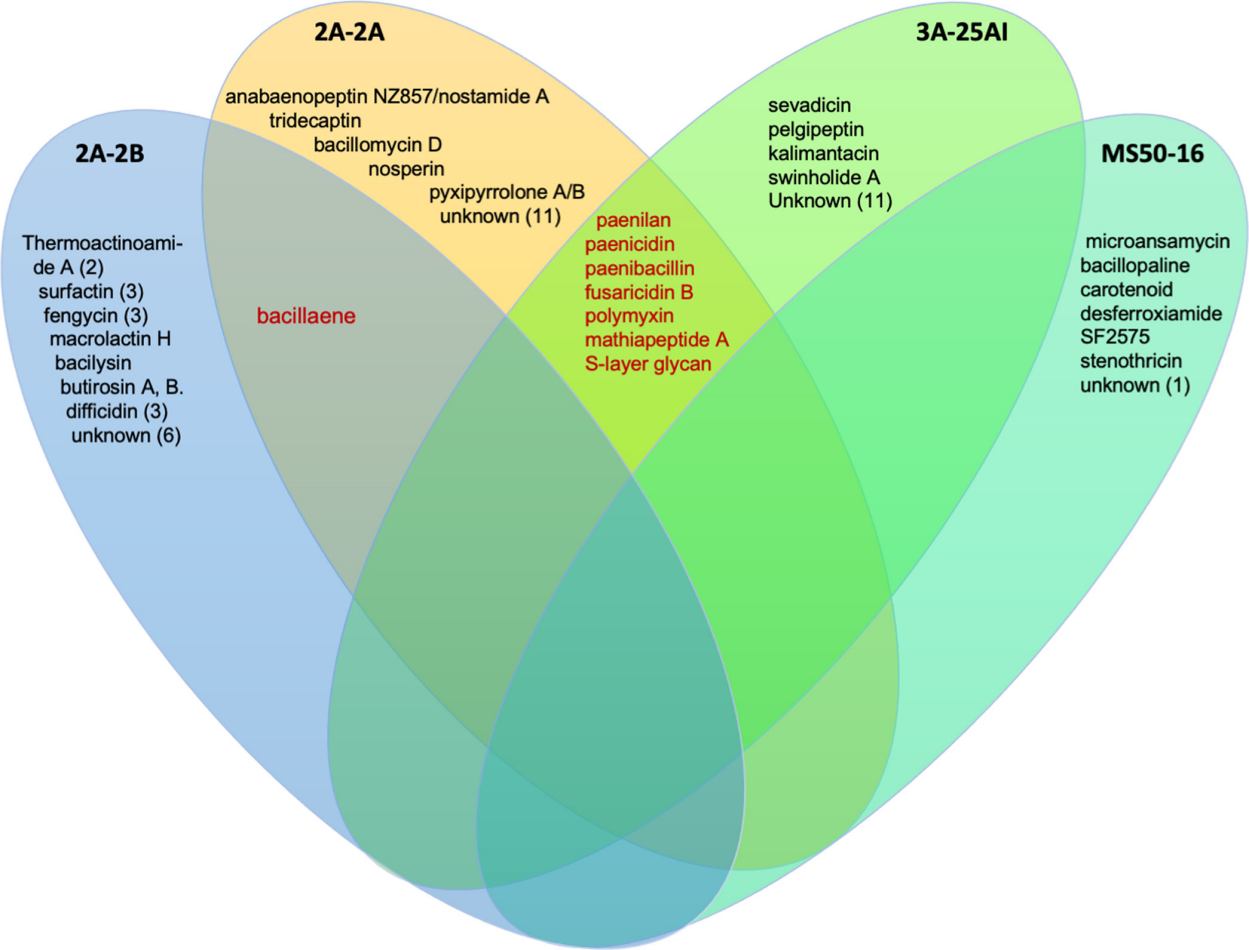
Antimicrobial secondary metabolite biosynthesis gene clusters (BGCs) in each of the four bacterial genomes studied, 2A-2B, 2A-2A, 3A-25AI, and MS50-16. With data obtained using the antiSMASH tool, antiSMASH 7 beta version.

However, *B. velezensis* 2A-2B showed notable divergence in the conserved gene clusters for the biosynthesis of antibiotic-like metabolites, in which no coincidence with the *P. polymyxa* 2A-2A and 3A-25AI was observed, except for one BGC for bacillaene biosynthesis, shared only with 2A-2A (Fig. 7 to 11).

For the MS50-16 genome, the antiSMASH analysis revealed only seven regions with BGCs, which were six clusters of genes with low similarity to known BGCs for the biosynthesis of desferroxyamine, carotenoid, stenothricin, bacillopaline, SF2575, and microansamycin, all with similarity to known BGCs between 4 and 50%, and a T3PKS-type gene cluster with no similarity to known BGC (Fig. 7 to 11). These seven regions with BGCs occupy 145 kb, which is 4.2% of the genome.

The 2A-2B genome was distinguished by having three BGCs for fengycin and three for surfactin antibiotics as follows: one BGC for fengycin with high similarity (80%) with respect to the known BGC for fengycin in the MIBiG database and the other two with similarity between 20 and 26%. For the other three BGCs for surfactin, the similarity was between 8 and 47% (Fig. 7). In 2A-2B, the three BGCs for fengycin biosynthesis were found to span 87.4-, 12.8-, and 9.4-kb regions, respectively. The longest comprises a candidate cluster and a protocluster that contains about 32 core genes. In the second BGC, a candidate cluster, the protocluster, and proto-core cluster are identified; there are also three core genes and more than 25 secondary genes. In the smaller BGCs in 2A-2B, it is found that the candidate cluster, the proto-core cluster, and protocluster, as well as two core genes and more than 20 genes with NRPS domains, are identified throughout the region (Fig. 8). Similarly, for surfactin, three BGCs are found in the 2A-2B genome, the first of them covering a region of 28.9 kb, the second with 15.0 kb, and the third with 8.9 kb. In the largest BGC, a candidate cluster is also identified, a protocluster, and contains 18 core genes. In the second BGC, the candidate cluster, the protocluster, and 14 core genes are distinguished, in addition to 25 or more secondary genes. Additionally, in the third BGC of 2A-2B, a candidate cluster, protocluster, and two core genes are identified, with more than 23 accompanying genes (Fig. 9).

### Secondary metabolite biosynthetic gene clusters identified using PRISM tool.

**FIG 12:**
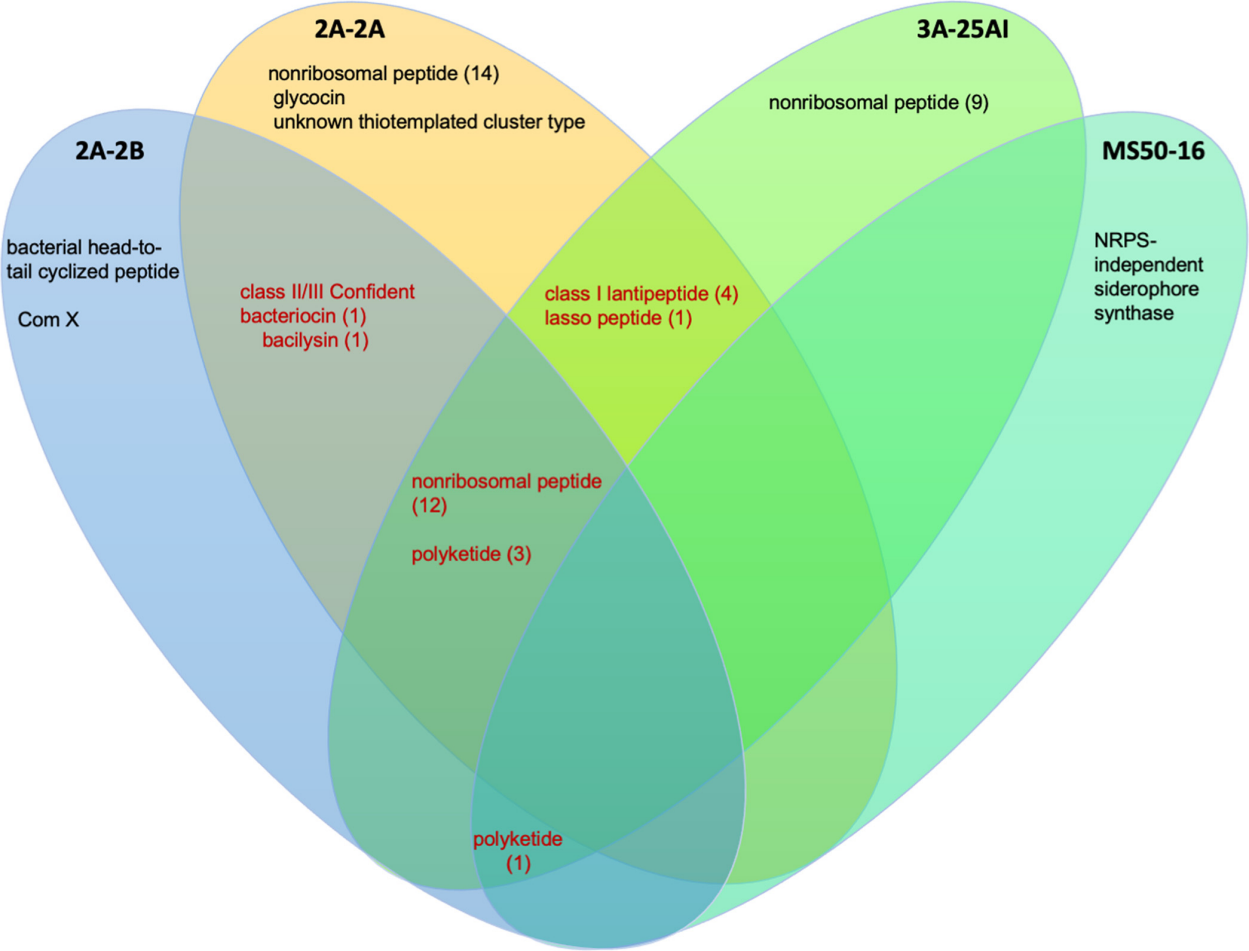
Antimicrobial secondary metabolite biosynthesis gene clusters (BGCs) in each of the four bacterial genomes studied, 2A-2B, 2A-2A, 3A-25AI, and MS50-16. With data obtained using the PRISM tool, version 4.

In the 2A-2B genome, 20 gene clusters for biosynthesis of secondary metabolites were identified. In these BGCs, 12 nonribosomal peptides, four polyketides, one class II/III confident bacteriocin, one bacilysin, one bacterial head-to-tail cyclized peptide, and one ComX were synthesized (Fig. 12).

In the 2A-2A genome, 38 gene clusters for biosynthesis of secondary metabolites were identified. In these BGCs, the secondary metabolites synthesized correspond to 26 nonribosomal peptides, four polyketides, four class I lantipeptides, one lasso peptide, one bacilysin, one class II/III confident bacteriocin, and an unknown thiotemplated cluster type (Fig. 12).

In the 3A-25AI genome, 30 gene clusters were identified, in these BGCs, the secondary metabolites synthesized correspond to 21 nonribosomal peptides, four polyketides, four class I lantipeptides, and one lasso peptide (Fig. 12).

Two gene clusters were identified in the MS50-16 genome, one NRPS-independent siderophore synthase and one polyketide, the latter shared with the other two bacterial strains (Fig. 12).

## DISCUSSION

### 2A-2B: outstanding inhibition of pathogens, friendly interaction in the root of chili pepper plant, and protection in plants infected with root pathogens.

Of the four bacteria tested in this study, *B. velezensis* 2A-2B stands out for its ability to inhibit the four phytopathogens, with an inhibition 18 to 30% higher against the three fungi and the oomycete than the two *Paenibacillus* strains (Fig. 1 and 2). At the same time, it showed avirulent, friendly behavior and was not disease-causing when inoculated in the root of the chili pepper plant (Fig. 3), a characteristic that prevails when the interaction occurred in plants in potted soil under bioclimatic chamber conditions. Conversely, 2A-2A and 3A-25AI showed hostility when inoculated in the root of the chili pepper plant (Fig. 3). These two characteristics of 2A-2B make it a valuable material to make a biotechnological tool to counteract root pathogens in chili pepper cultivation. Strains 2A-2A and 3A-25AI, by themselves, showed much less promise for the purpose of inhibiting these pathogens and were even counterproductive in relation to the interaction with the root of the plant.

Remarkably good performance was observed in 2A-2B when preinoculated into pepper plant roots that were then infected with virulent strains of *P. capsici* and R. solani. The disease index in plants preinoculated with bacteria dropped to 0.6 but was 3.7 in plants infected by pathogens without the presence of strain 2A-2B bacteria. Similar to the *in vitro* studies, the studies in pots with soil in a bioclimatic chamber, at 4 days postinoculation, confirmed that 2A-2B maintained its ability to inhibit pathogens and protect the chili pepper plant (Fig. 4). This is a rhizobacteria with potential as a biocontrol agent against root pathogens, at least in chili cultivation.

### Genome sequencing identified the bacteria as *B. velezensis*, *P. polymyxa*, and *K. polaris*, with a different evolutionary trajectory in *B. velezensis*.

Once assembled, the size of the genomes fluctuated between 3.4 and 5.7 MB. In the alignment of genomes in the database for microbial genomes in the NCBI server, these corresponded in size with the species to which they showed greater identity and similarity. With identities above 98%, *B. velezensis* and *P. polymyxa* corresponded to 2A-2B as well as 2A-2A and 3A-25AI, respectively; meanwhile MS50-16 had less than 85% identity to *K. polaris*. These results were confirmed using the ANI. GC content was between 46 and 45.5% for *B. velezensis* and *P. polymyxa*, respectively, but was highest in *K. polaris* (65.5%). It is known that GC content in bacterial genome can vary between 13 and 75% (14) and that this is closely related in part to the environmental factors where the bacterium grows (15). The bacteria in this study came from soil and rhizosphere; rhizosphere samples were obtained from cultivated and wild plants from Zacatecas state in the central north region of Mexico where the climate is dry and semiwarm (BWhu). The wild plant sampled species was Sporobolus airoides (Torr.) Torr. Prokaryotes in high-temperature environments have higher GC content than those in low temperatures, suggesting that higher GC contents may be related to thermal adaptation (16). Mutation events in the DNA, ecological niche conditions, and above all, horizontal gene transfer affect the GC content in bacterial genomes (17). For the bacteria in this study, the distant signatures between species and the percentages of GC content in their genomes also seem to be a genetic characteristic of these species.

In the alignment of *B. velezensis* 2A-2B and *B. velezensis* JS25R as the reference genome in Mauve tool, there were 24 LCBs. Eleven LCBs in the 2A-2B genome were inverted and located in reverse complement orientation with respect to the reference genome (NZ_CP009679.1). In the multiple alignment analysis of genomes, to observe the level of conservation or divergence in 2A-2B with respect to the other three genomes of *B. velezensis* strains, 2A-2B had a high level of divergence in the structuring of the genome with respect to the other genomes of the same species. These divergence data in the structural arrangement indicated a somewhat different evolutionary trajectory in the 2A-2B genome. This result is not unusual given that these strains were isolated from spatially distant origins and had to adapt to very different habitat conditions. JS25R is from a Jiangsu province in China, GFP-2 was isolated from the intestine of Chiloscyllium plagiosum (Whitespotted bamboo shark) at the Zhejiang Sci-Tech University in China (18), and AD8 was isolated from a typical high-salt red pepper paste in China (19), while the 2A-2B strain originates from rhizosphere in the north central part of Mexico.

### Profile of distant BGCs between bacteria and relationship with phenotype: according to antiSMASH, 10 BGCs in *B. velezensis* 2A-2B make a difference.

The number and specificities of gene cluster for antibiotic secondary metabolite biosynthesis were revealed with antiSMASH. Conserved and divergent BGCs were clearly observed among the genomes of the four bacterial strains in this study. Only two bacterial strains, 3A-25AI and 2A-2A shared eight BGCs involved in the biosynthesis of paenicidin A, polymyxin B, paenibacillin, fusaricidin B, paenibacidin, martiapeptide A, paenilan, and S-layer glycan (Fig. 7). Particularly, the *B. velezensis* 2A-2B genome has 11 BGCs that synthesize the antibiotics surfactin (three BGCs), fengycin (three BGCs), macrolactin H, bacilysin, butyrosin A/butyrosin B, bacillaene, and difficidin (two BGCs); the bacillaene antibiotic was only shared with 2A-2A. Each bacterium had 4 to 10 clusters of genes in its genome for antibiotic biosynthesis that were specific for each bacterial strain (Fig. 7).

Considering this scenario of gene clusters for antibiotic biosynthesis in the genome of these four bacteria, and the phenotype of each strain in relation to the inhibition of four root pathogens in chili pepper, where *B. velezensis* 2A-2B stands out notably, we hypothesized that the great antifungal and antioomycete capacities of this bacterial strain depend on the antibiotics produced, specifically surfactin and fengycin, three BGCs for the biosynthesis of each of these antibiotics. Studies in sugarcane suggest that surfactin produced by Bacillus subtilis is one of the antifungal lipopeptides responsible for inhibiting 10 phytopathogenic fungi in this crop (20). B. subtilis SG6 was reported to have notable antifungal activity, which reduces kernel head blight caused by Fusarium graminearum. This antifungal activity is associated with the production of fengycin and surfactin (21). Fengycin purified from crude extract derived from Bacillus amyloliquefaciens FZB42 culture, applied on F. graminearum, was shown to result in structural deformation of hyphae, and in a plant experiment, fengycin significantly suppressed F. graminearum growth (22). These data coincided partially with the results of BGCs that we found in *B. velezensis* 2A-2B. In another genomic sequencing and mining study, *B. velezensis* CC09 showed high potential for biocontrol of fungal phytopathogens. Thirteen secondary metabolite-encoding clusters were identified, and these clusters of genes had several genetic sequences responsible for the biosynthesis of surfactin, bacillaene, macrolactin, among others (23). These data also closely agreed with our results in *B. velezensis* 2A-2B. The three BGCs for surfactin biosynthesis in 2A-2B showed 52, 39, and 8% similarity with respect to the reference BGCs in *B. velezensis* FZB42 from the GenBank (Fig. 9); the same was observed with the three BGCs for fengycin (Fig. 8). Surfactin is a lipopeptide involved in the formation of biofilm and colonization of plant roots (24), with a polar amino acid head acting as the biosurfactant that produces cationic channels on lipid membrane bilayers and contributes to its antifungal properties (25, 26). The biosynthesis of surfactin is favored when bacteria grow on root exudates. Surfactin is a key component in *B. velezensis* and B. subtilis, which are used in the developmental process of biofilm formation, motility, and early colonization (27–29). It has been reported that bacteria also qualitatively modulate the pattern of homologous surfactins coproduced in plants and form major variants that are the most active in triggering plant immunity. Thus, surfactin has shared benefits since it reinforces the defensive capacity of the host (30). Furthermore, fengycin provides remarkable antagonistic activity against filamentous fungi; in addition, surfactin and fengycin can act synergistically amplifying their respective biological functions (31).

Although *P. polymyxa* 2A-2A and 3A-25AI had an arsenal of 12 BGCs for the biosynthesis of the same number of antibiotics, 8 shared and 9 (5 in 2A-2A and 4 in 3A-25AI) divergent between them, these data suggest that this is not enough for them to develop an effective inhibitory effect against the four phytopathogens included in this study, and although these bacterial strains have BGCs for the biosynthesis of antibiotics, such as fusaricidin, which is active against a broad array of phytopathogenic fungi (32), this seems to indicate that one or more of the gene clusters for the antibiotic biosynthesis that 2A-2B possesses, particularly with antifungal action, are missing. For its part, MS50-16, which only had six known gene clusters for antibiotic biosynthesis with none shared with the other three bacteria, is devoid of these genes and the properties of antibiosis against other microorganisms. It is noteworthy that MS50-16 has a cluster of genes for the biosynthesis of a siderophore, as is the cluster for desferrioxamine (Fig. 7).

### *B. velezensis* 2A-2B had the most diverse arsenal of BGCs for antibiotics identified using PRISM tool.

The data of gene clusters for the biosynthesis of antibiotic secondary metabolites analyzed with the PRISM tool revealed conserved and divergent gene clusters among the genomes of the four bacterial strains in this study. 2A-2A, 2A-2B, and 3A-25AI shared 12 BGCs of the nonribosomal peptide biosynthesis type and 4 of the polyketide type (Fig. 8). *P. polymyxa* 2A-2A and 3A-25AI also had other BGCs for the biosynthesis of the nonribosomal peptides type, four for 2A-2A and seven for 3A-25AI. Clusters of genes not shared with the other bacterial strains were found in the 2A-2B genome; these clusters encode for the biosynthesis of bacilysin, bacterial head-to-tail cycled peptide, ComX, and class II/III confident bacteriocin. Meanwhile, the *K. polaris* MS50-16 genome had one BGC shared with the other three bacterial strains that codes for polyketide biosynthesis and had a specific nonshared gene cluster that codes for NRPS-independent siderophore synthase. 2A-2A, 2A-2B, and 3A-25AI had greater numbers of BGCs for the biosynthesis of antibiotic secondary metabolites, each had 21 to 23 clusters, while *K. polaris* MS50-16 only had two BGCs (Fig. 8). Polyketides and lipopeptides with their own PKS and NRPS are common in plant-associated bacteria, prominently in bacteria of the genera *Bacillus* and *Paenibacillus* (33). This is consistent with our results for 2A-2B as well as 2A-2A and 3A-25AI, which belong to *Bacillus* and *Paenibacillus*, respectively. The three strains are rhizobacteria; interestingly, other bacterial genera that live in other niches, not in plant hosts, rarely have these groups of genes. This reinforces the idea of an important function of these secondary metabolites in rhizobacteria (33).

These results of BGCs for antibiotics identified with PRISM suggest that the greater inhibition capacity of 2A-2B against the four phytopathogens and its friendly interaction in chili pepper plant roots are because of the gene clusters for biosynthesis of bacilysin, for head-to-tail cyclized peptide, or for ComX, which are specific for 2A-2B. The bacilysin peptide can also activate induced systemic resistance in plant through the positive regulation of *erf1* for the ethylene signaling pathway, which in turn is related to higher expression of *hel*, which is a defense gene (34, 35). Cyclic peptides produced as secondary metabolites in bacteria are antimicrobial compounds synthesized by NRPS that target polymers, such as chitin and glucans, that comprise the cell wall in fungi (36). ComX is a pheromone peptide that regulates the expression of a cluster of four *srfA* open reading fames (ORFs) that are responsible for the biosynthesis of surfactin (37); this reinforces the aforementioned finding of three BGCs for surfactin biosynthesis found with the antiSMASH tool. Plant-associated strains of *B. amyloliquefaciens* have a high number of clusters of genes related to the biosynthesis of nonribosomal peptides (38). This characteristic appears to be present in the strains of *B. velezensis* and *P. polymyxa* but not in *K. polaris* included in the present study; and indeed, these strains are plant-associated since they came from the rhizosphere of wild or cultivated plants (39, 40). *K. polaris* MS50-16, as revealed using antiSMASH and PRISM, was devoid of gene clusters for secondary metabolites with antibacterial potential.

Thus, *B. velezensis* 2A-2B had a greater diversity of BGCs for the biosynthesis of nonribosomal peptides, polyketides, lanthipeptides, and also for another three different antibiotics; it also had possibly the greatest inhibition capacity against phytopathogens due to the BGCs for nonribosomal peptides and lanthipeptides, since the *P. polymyxa* strains also possess a number of these BGCs, and these strains showed less inhibition capacity against the phytopathogens in this study. Furthermore, the genetic divergences in BGC content between *P. polymyxa* 2A-2A and 3A-25AI, even given the 99.96% genomic identity between them, indicates that the diversification between these two bacterial strains has been happening over time in the biosynthesis function of antibiotics to face other competing microorganisms, and said biological function is encoded in 0.04% of the genome in which these strains differ.

### Conclusions.

In the four bacteria studied here, strain 2A-2B was prominent as a biocontrol agent to impede root pathogens in chili pepper crops and has friendly interaction at the root of the plant. In the sequenced genomes of these four rhizobacteria, we determined that 2A-2A and 3A-25AI both belonged to *P. polymyxa* with high genomic similarity between them, MS50-16 with less identity was a strain of *K. polaris*, and 2A-2B was confirmed to be a strain of *B. velezensis*. *B. velezensis* 2A-2B holds conserved regions throughout the genome, without regions lacking homology compared with the reference genome, but a different evolutionary trajectory is observed compared to three reference genomes of the same species.

In addition, 2A-2B was characterized by having 13 gene clusters for the antimicrobial metabolite biosynthesis not shared with the other three bacteria, including surfactin (three BGCs), fengycin (three BGCs), and difficidin (three BGCs). These BGCs, especially those related to the biosynthesis of surfactin and fengycin (six BGCs), hypothetically, may well have a relevant function in the production of antibiotics that provided the greatest inhibition capacity against the phytopathogens studied herein. We cannot rule out that some of the BGCs of the NRPS, PKS, or other unknown BGCs identified in 2A-2B may be important. *P. polymyxa* 2A-2A and 3A-25AI had a greater number of gene clusters for antimicrobial metabolite biosynthesis, particularly NRPS; however, these clusters are not enough to efficiently inhibit the four phytopathogens in chili pepper roots. MS50-16 had diminished antifungal potential due to the low number of BGCs for antimicrobial metabolites. Fusaricidin B in 2A-2A and 3A-25AI seemed to be an important antibiotic for phytopathogenic fungi inhibition. Surfactin (three BGCs) in 2A-2B contributed to the friendly interaction with the plant given its relevant function already reported for biofilm formation, motility, and root colonization in addition to reinforcing the plant’s defense against pathogens. Therefore, *B. velezensis* 2A-2B has high potential for biocontrol of root pathogens in chili pepper and other vegetable crops. Further sequenced genome analysis is required to delve deeper into understanding genetic characteristics related to the inhibition of phytopathogens and interaction with the plant.

## MATERIALS AND METHODS

### *In vitro* growth conditions of bacteria, confrontation with phytopathogens, and interaction in plant roots.

The bacteria studied here were collected from a soil sample for agricultural use or from the rhizosphere of a wild plant. The strain MS-5016 was obtained from soil and the strains 3A-25AI, 2A-2A, and 2A-2B come from the rhizosphere of *Sporobolus airoides* (Torr.). The location is the agricultural and forestry area of the municipality of Morelos, state of Zacatecas, Mexico. These bacteria grow well in LB, KB, TSA, and PDA media with an optimum temperature of 28°C.

Each of the bacterial strains and the pathogenic fungi (F. solani, F. oxysporum, R. solani) or the oomycete (*P. capsici*) were confronted *in vitro* on plate with PDA, TSA, or KB medium. Each bacterial strain was inoculated at 4 equidistant points on a plate with culture medium, incubated for 2 days at 28°C, and immediately a mycelial plug of 4 mm from the edge of the colony with fresh growth (5 day old) of *P. capsici* or 2-day-old cultures of the three fungi was placed in each plate in the center, 5 cm from the growth point of the bacteria, with an *n* value of 12. The percent inhibition of pathogens was determined, and the inhibitory effect was calculated according to the following formula: inhibitory effect (%) = [(R – r)/R] × 100.

R and r indicate the radius of pathogen mycelial growth in control and treatment, respectively. Assay results in PDA medium were statistically analyzed. Data were transformed to square root arcsine, and one-way analysis of variance (ANOVA) was performed.

For the plant-bacteria interaction tests, 12-days-old Mirasol cultivar chili seedlings grown *in vitro* were immersed by the root for 10 min in a bacterial suspension with an optical density at 610 nm adjusted to 0.1 and bacteria previously grown in LB medium for 24 h. Immediately, the plants were transferred to a plate with 50% MS medium, half of the plate without MS medium, and in vertical position; the root of the plants anchored on MS medium and foliage in the upper middle part without culture medium. The plants on the plates were placed in a bioclimatic chamber with a 14-h light photoperiod at 27°C. Control plants without bacterial inoculation were included. Each treatment was performed in quadruplicate, and each plant was considered a replicate. The evolution of tissue necrosis and wilted leaves (diseased plant) or healthy root and plants (asymptomatic) was recorded for 10 days. Disease symptoms were expressed as a rapidly expanding dark brown lesion at the base of the stem or along the entire length of the roots, in addition to wilting of the leaves. Disease severity was scored at 7 days after root dip inoculation according to the following criteria: 0, asymptomatic; 1, minor symptoms with less than 5% affected roots and 15% or less of wilted leaves; 2, moderate symptoms with 5.1% to 20% affected roots and 15.1% to 50% wilted leaves; 3, severe symptoms with 20.1% or more of affected roots and 50.1 to 80% wilted leaves; and 4, dead plants. Values of disease severity between 0 and 1 were considered healthy plants, and values between 2 and 4 were considered diseased plant. Disease severity index was calculated. DSI = ∑ (disease score × number of plants with that disease score)/total number of plants. ANOVA and Tukey test was performed.

### Coinoculation in plant root with strain 2A-2B bacteria and pathogens *P. capsici* and R. solani under bioclimatic chamber conditions.

Sixteen-day-old pepper plants grown *in vitro* were inoculated by submerging the root in bacterial suspension as previously described and then potted with sterile soil, soil prepared with peat (Peat-Moss), loamy-clay native soil, and vermiculite in proportions of 40-40-10%. After being established in a pot, it was inoculated with wheat grains infected and well colonized with *P. capsici* or R. solani pathogens. The inoculation of both pathogens was simultaneous. One infected wheat grain was placed 0.5 cm away from the base of the plant and 1 cm deep in the soil. Immediately, the potted plants were taken to a bioclimatic chamber with a 14-h light photoperiod at 27°C. Control plants without bacteria were included. Each treatment was performed in quadruplicate, and each plant was considered a replicate. The evolution of diseased or healthy plants was recorded for 4 days. Symptom values and disease progression were the same as previously described. Analysis of variance and Tukey’s test was performed.

### Extraction of genomic DNA from bacterial strains and preparation of DNA libraries.

A colony sample of each bacterial strain was inoculated in 5 mL of LB liquid medium and stirred at 150 rpm for 24 h at 28°C. Bacterial cultures were centrifuged at 4,000 × *g*, and genomic DNA extraction was performed on the bacteria cell pellets. A protocol based on cetyltrimethylammonium bromide (CTAB) reagent was used for DNA extraction (41). The DNA was analyzed by 1% agarose gel electrophoresis and quantified in a Qubit 2.0 fluorometer (Invitrogen). The preparation of libraries for sequencing was following the instructions of the Nextera kit (Illumina, San Diego, CA, USA). One nanogram of genomic DNA was diluted in 5 μL of water, enzymatically fragmented, and tagged by PCR. The size and distribution of fragments in the libraries was verified in a bioanalyzer (2100 Bioanalyzer; Agilent Technologies). From these libraries, after standard normalization, 15 pM were used for genome sequencing.

### Genome sequencing and assembly.

For high-throughput genomic sequencing, the MiSeq platform was used under a protocol of sequencing by synthesis (MiSeq; Illumina). Microfluidics-based automated electrophoresis in bioanalyzer equipment (Agilent Bioanalyzer) made it possible to observe and ensure the quality of the genomic DNA libraries that would be sequenced, yielding sufficient quality for the genomic DNA libraries of the 4 bacterial strains. This part of the work was carried out in the Sequencing laboratory in the Biological Sciences Unit of the Autonomous University of Zacatecas, Mexico. After sequencing, the reads were cleaned using Trimmomatic to remove sequences of Illumina adapters and ensure average quality (42). Then, the quality of read was analyzed in FastQC (43) using a lower threshold for contig length of 200 bp. The assembly of genomes was carried out using SPAdes (Galaxy Version 3.15.3+galaxy2) in the Galaxy platform (10) and in QUAST 4.1 (44). The quality of assembles was analyzed including reference genome. The circular map of the genomes was generated using CGView software (45). Genome annotation was done with the Prokka tool (Galaxy Version 1.14.6+galaxy1) in the Galaxy platform (11), with default parameters including improved gene predictions for highly fragmented genomes, fast mode, similarity e-value cut-off 1e−06 and searching for noncoding RNAs (ncRNAs).

### Alignment in prokaryote genome database and average nucleotide identity calculation to define bacterial species.

The sequenced genome obtained for each of the bacteria was aligned in the database for prokaryotic genome sequences to perform phylogenetic analysis and define possible species of bacteria based on similarity percentages. BLAST algorithm for bacterial genomes optimized for highly similar sequences (megablast) was used in the whole-genome database on the NCBI server. Average nucleotide identity (ANI) (46) was also calculated to confirm and define the species of each bacterial isolate under study. For ANI analysis, reference genomes *B. velezensis* JS25R (NZ_CP009679.1) and *P. polymyxa* ZF129 (NZ_CP040829.1) were included.

### Strain 2A-2B genome structuring analysis by species genome alignment.

In order to analyze the structuring and possible rearrangements in the genome of strain 2A-2B, an alignment of the 2A-2B genome was carried out with the reference genome obtained from the NCBI gene bank, which showed the greatest coverage in the alignment. This exercise allowed us to observe local blocks of collinearity, regions of homology throughout the genome, as well as the forward or reverse orientation of the LCBs in strain 2A-2B. Multiple alignment was also performed to analyze the level of conservation or the divergence in the structuring of the genomes between strain 2A-2B and three other genomes of the same species of *B. velezensis*. These analyses were performed using the Mauve tool version snapshot 2015-02-25 (47).

### Bioinformatic analysis for biosynthetic gene clusters of secondary metabolites.

Using the antiSMASH tool, each bacterial genome was analyzed for clusters of genes involved in the biosynthesis of secondary metabolites, including antibiotics. This software takes full advantage of several tools that have been developed previously such as KnownClusterBlast, MIBiG cluster comparison, Cluster Pfam analysis, ActiveSiteFinder, Pfam-based GO term annotation, and others. The antiSMASH analysis capacity is enhanced with other open access tools, such as NCBI BLAST, HMMer 3, Muscle 3, FastFree, and PySVG, among others. antiSMASH is perhaps the most widely used tool today to detect and characterize clusters of biosynthetic genes in bacteria and fungi (48, 49). The antiSMASH 7 beta version was used (49). For this task, the strict detection astringency parameter was applied, and the extra characteristics of Known Cluster BLAST, MIBiG cluster comparison, Cluster Pfam analysis, ClusterBlast, ActiveSiteFinder, Pfam-based GO term annotation, SubClusterBlast, RREFinder, and TIGRFam analysis were used. To analyze the similarity and structure of the BGCs of the bacterial strains under study in comparison with the reference BGCs in the BGC repository on the online MIBiG server (https://mibig.secondarymetabolites.org), the presence and number of core biosynthetic genes, additional biosynthetic genes, and other regulatory genes were reviewed.

Another tool for this same purpose is called PRISM (prediction informatics for secondary metabolomes), which is an algorithm for predicting secondary metabolite structures and their biological activity from microbial genome sequences. The PRISM platform makes it possible to decipher the information in genomic sequences with chemical structure of the encoded molecules, thus allowing the search for genetically encoded natural products in genomes (50). PRISM version 4 was used.

### Data availability.

The Whole Genome Shotgun projects have been deposited at DDBJ/ENA/GenBank under the accession numbers MLCV00000000, JALGWU000000000, MLCZ00000000, and MLDA00000000, and the versions described in this paper are versions MLCV01000000, JALGWU010000000, MLCZ01000000, and MLDA01000000 for the bacterial strains 2A-2B, 2A-2A, 3A-25AI, and MS50-16, respectively.
